# Impact of AI system on recognition for anatomical landmarks related to reducing bile duct injury during laparoscopic cholecystectomy

**DOI:** 10.1007/s00464-023-10224-5

**Published:** 2023-06-26

**Authors:** Yuichi Endo, Tatsushi Tokuyasu, Yasuhisa Mori, Koji Asai, Akiko Umezawa, Masahiro Kawamura, Atsuro Fujinaga, Aika Ejima, Misako Kimura, Masafumi Inomata

**Affiliations:** 1 Department of Gastroenterological and Pediatric Surgery, Faculty of Medicine, Oita University, Oita, Japan; 2 Department of Information System and Engineering, Faculty of Information Engineering, Fukuoka Institute of Technology, Fukuoka, Japan; 3 Department of Surgery 1, School of Medicine, University of Occupational and Environmental Health, Kitakyushu, Fukuoka Japan; 4 Department of Surgery, Toho University Ohashi Medical Center, Tokyo, Japan; 5 Minimally Invasive Surgery Center, Yotsuya Medical Cube, Tokyo, Japan

**Keywords:** Artificial intelligence (AI), Anatomical landmarks, Bile duct injury (BDI), And laparoscopic cholecystectomy (LC)

## Abstract

**Background:**

According to the National Clinical Database of Japan, the incidence of bile duct injury (BDI) during laparoscopic cholecystectomy has hovered around 0.4% for the last 10 years and has not declined. On the other hand, it has been found that about 60% of BDI occurrences are due to misidentifying anatomical landmarks. However, the authors developed an artificial intelligence (AI) system that gave intraoperative data to recognize the extrahepatic bile duct (EHBD), cystic duct (CD), inferior border of liver S4 (S4), and Rouviere sulcus (RS). The purpose of this research was to evaluate how the AI system affects landmark identification.

**Methods:**

We prepared a 20-s intraoperative video before the serosal incision of Calot’s triangle dissection and created a short video with landmarks overwritten by AI. The landmarks were defined as landmark (LM)-EHBD, LM-CD, LM-RS, and LM-S4. Four beginners and four experts were recruited as subjects. After viewing a 20-s intraoperative video, subjects annotated the LM-EHBD and LM-CD. Then, a short video is shown with the AI overwriting landmark instructions; if there is a change in each perspective, the annotation is changed. The subjects answered a three-point scale questionnaire to clarify whether the AI teaching data advanced their confidence in verifying the LM-RS and LM-S4. Four external evaluation committee members investigated the clinical importance.

**Results:**

In 43 of 160 (26.9%) images, the subjects transformed their annotations. Annotation changes were primarily observed in the gallbladder line of the LM-EHBD and LM-CD, and 70% of these shifts were considered safer changes. The AI-based teaching data encouraged both beginners and experts to affirm the LM-RS and LM-S4.

**Conclusion:**

The AI system provided significant awareness to beginners and experts and prompted them to identify anatomical landmarks linked to reducing BDI.

**Supplementary Information:**

The online version contains supplementary material available at 10.1007/s00464-023-10224-5.

Laparoscopic cholecystectomy (LC) is a standardized surgical procedure for cholelithiasis and acute cholecystitis [1]. Although robotic surgery has grown in popularity in recent years, the demand for LC remains high, and it is expected that the annual number of cases performed will continue to rise in the future due to the increase in elderly patients [2]. A clinically important issue for LC is avoiding bile duct injury (BDI), a severe intraoperative complication of LC. However, even with the advancements in techniques, the incidence of BDI was about 0.4%, with no evidence of decline [3].

Way et al. analyzed the cause of BDI and verified that about 97% of BDI was due to human visual error [4]. Iwashita et al. performed a questionnaire survey of hepato-biliary-pancreatic professionals in Japan, South Korea, and Thailand, and the results showed that approximately 60% of BDIs were caused by misidentifying anatomical landmarks in the Calot’s triangle dissection [5]. However, the authors presented an artificial intelligence (AI) system for advanced intraoperative learning of the extrahepatic bile duct (EHBD), cystic duct (CD), the lesser edge of liver S4 (S4), and the Rouviere sulcus (RS) as anatomical landmarks before serosal incision of Calot’s triangle dissection [6].

However, because EHBD and CD run beneath the serosa and adipose tissue, they cannot be seen directly, but experienced doctors can identify the course of the EHBD and CD by looking at the serosa and adipose tissue in the surgical field. Therefore, several studies to prevent BDI have been reported [7–9], and BDI most often occurs due to the misrecognition of the EHBD as the CD before and during Calot’s triangle dissection [4, 10].

The authors have developed an intraoperative system that can detect anatomical landmarks in real-time during cholecystectomy procedures and have completed verification of their device through a single-center clinical feasibility trial (J-SUMMIT C-01) [11]. However, there are no reports verifying how AI affects the identification of anatomical landmarks.

The purpose of this research was to evaluate how the AI system affects anatomical landmark identification.

## Materials and methods

### Deep learning model for landmark indication

The authors collected 230 videos of LC conducted from 2019 to 2021 in Oita University Hospital, and 95 cases that remained on video and had mild inflammation were selected to construct our deep learning model for landmark indication, where only sixteen cases were classified as having mild acute cholecystitis. We excluded LC cases with severe inflammation and abnormal biliary anatomy because it was difficult to annotate anatomical landmarks.

Since BDI due to anatomical misidentification occurs during the operation after serosotomy in addition to RS and S4 and CD and EHBD, which are covered with the fat of the hepatoduodenal ligament, were defined as the anatomical landmarks in this study.

EHBD and CD were annotated with their respective strikes on the serosal surface and classified as landmarks in this study: landmark (LM)-EHBD and LM-CD [11].

We asked the participants to draw the common bile duct and common hepatic bile duct together when they annotated the landmarks; thus, the CBD labeling in previous research was changed to EHBD in this research.

A total of 1754 still images were manually extracted from Calot triangle dissection scenes from surgical videos of 95 cases, automatically selecting frames at 1 fps and further selecting images in which one of the landmarks fit within the frame.

A surgeon certified by JSES annotated 1610 images of LM-EHBD, 1503 images of LM-EHBD, 1623 images of LM-S4, and 1505 images of LM-RS. Of these, 76 cases were used as training data, and the remaining 19 cases were used as test data to evaluate the performance of the learning model.

We employed YOLOv3, an object detection algorithm dependent on deep learning, as AI. When we started building our learning model, YOLOv3 was state-of-the-art in object detection. We chose YOLOv3 because it is coded in C, which allows for fast processing, and because, from experience, it is also a good match for anatomy detection in intra-abdominal images. To show the teaching data of landmarks along the anatomical structure, we learned them as the same label after dividing the annotation data into 16 × 16 pixels. In addition, to express the ambiguity between LM-CD and LM-EHBD, transparency was given to the display color of the rectangle according to the confidence level of AI per the formula (1). For the display color of the rectangle, we gave transparency according to the probability of AI, as described by the formula (1), where *a* = 0.75 and *b* = 0.25 were given by the authors empirically.

1 $${\text{Transparency }} = \, a{\text{ Probability }} + \, b$$

We applied the YOLOv3 learning model to the test datasets of 19 cases, comprising 190 images of LM-EHBD, 186 images of LM-EHBD, 192 images of LM-S4, and 190 images of LM-RS. The average Dice coefficient for each landmark were LM-EHBD: 0.526, LM-CD: 0.208, LM-S4: 0.353, and LM-RS: 0.211. The Dice coefficient is one of the indices used to evaluate the AI performance of image recognition, and the closer it is to 1, the higher the accuracy.

### Materials

This study investigated the operator’s LM-EHBD and LM-CD before and after learning the landmarks to assess whether the landmarks on endoscopic images corrected the operator’s visual misperceptions. We concentrated on the change in perspective. We collected 500 LC cases performed at other institutions where the authors do not belong and randomly extracted videos of 20 cases. In preparation for recognition before Calot’s triangle dissection, a frame was selected before the start of the incision, and 20-s short videos were formed. After Calot triangle dissection, supporting tissue around CD and EHBD was removed, and the running of the CD and EHBD were expected to be considerably different. Therefore, it was deemed difficult to verify the running of the CD and the EHBD before and after the Calot triangle dissection. In this study, four EEC were asked to discuss one image each, for a total of 20 images, and the area in which the four EEC agreed was the Ground truth. The EEC created the correct data for the selected 20 images through these processes.

### Protocol

We formed videos overwritten with landmark data by adding our landmark teaching AI to a 20-s short video (supplement 1, movie). Four beginners and four experts were recruited as subjects for this experiment. In this study, a doctor with less than fifty cases of LC surgery under their belt was considered a beginner. The expert was defined as JSES board-certified doctors. The difficulty of recognizing the LM-EHBD and the LM-CD was indicated on a 3-point scale questionnaire. Following that, subjects watched the video that had been overwritten with landmark information, and if there was a change in each perspective, the annotation was corrected (Phase B). The subjects were asked to answer a three-point scale of whether or not the landmark information urged them to identify the LM-RS and LM-S4.

### Evaluation

A questionnaire survey was performed to check whether there was any change in each subject’s perspective after viewing the AI-instructed video. Four EEC members contemplated the clinical importance of whether each change in perspective has changed to a safer or more critical side from the perspective of a safe first serosal incision before Calot’s triangle dissection.

The first serosal incision was determined by the relationship of the LM-S4, LM-RS, and LM-EHBD, and a safe transition was defined as shifting the LM-EHBD to the gallbladder portion. A critical transition was defined as shifting the LM-EHBD to the opposite side of the gallbladder. The EEC and subjects consented to this study and did not directly participate in evaluating the study results.

None of the subjects were involved in developing this research; however, they were provided an explanation of the research purpose, agreed to participate in the experiment, and provided informed consent. This study has been approved by the Ethics Committee of Oita University Hospital (# 2280-C28).

## Results

**Fig. 1:**
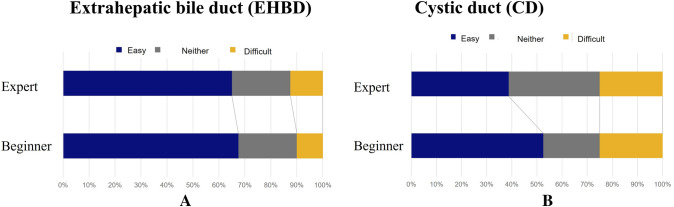
Results of the questionnaire survey (Phase A). The difficulty of recognizing the LM-EHBD (**A**) and the LM-CD (**B**)

The beginners and experts reported it was more challenging to recognize the LM-CD than the LM-EHBD. Furthermore, the experts tended to find it more challenging to recognize the LM-CD than the starters (Phase A, Fig. 1A, B).

**Fig. 2:**
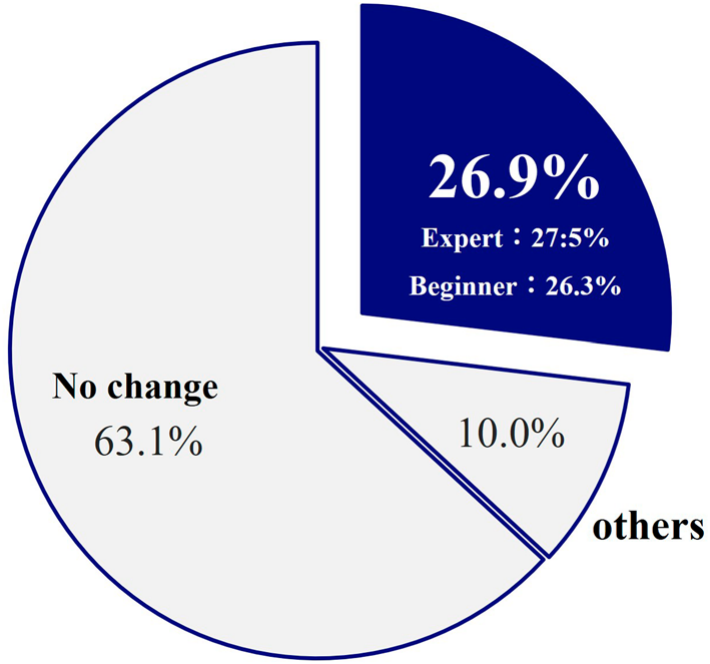
Percentage of cases with change

**Fig. 3:**
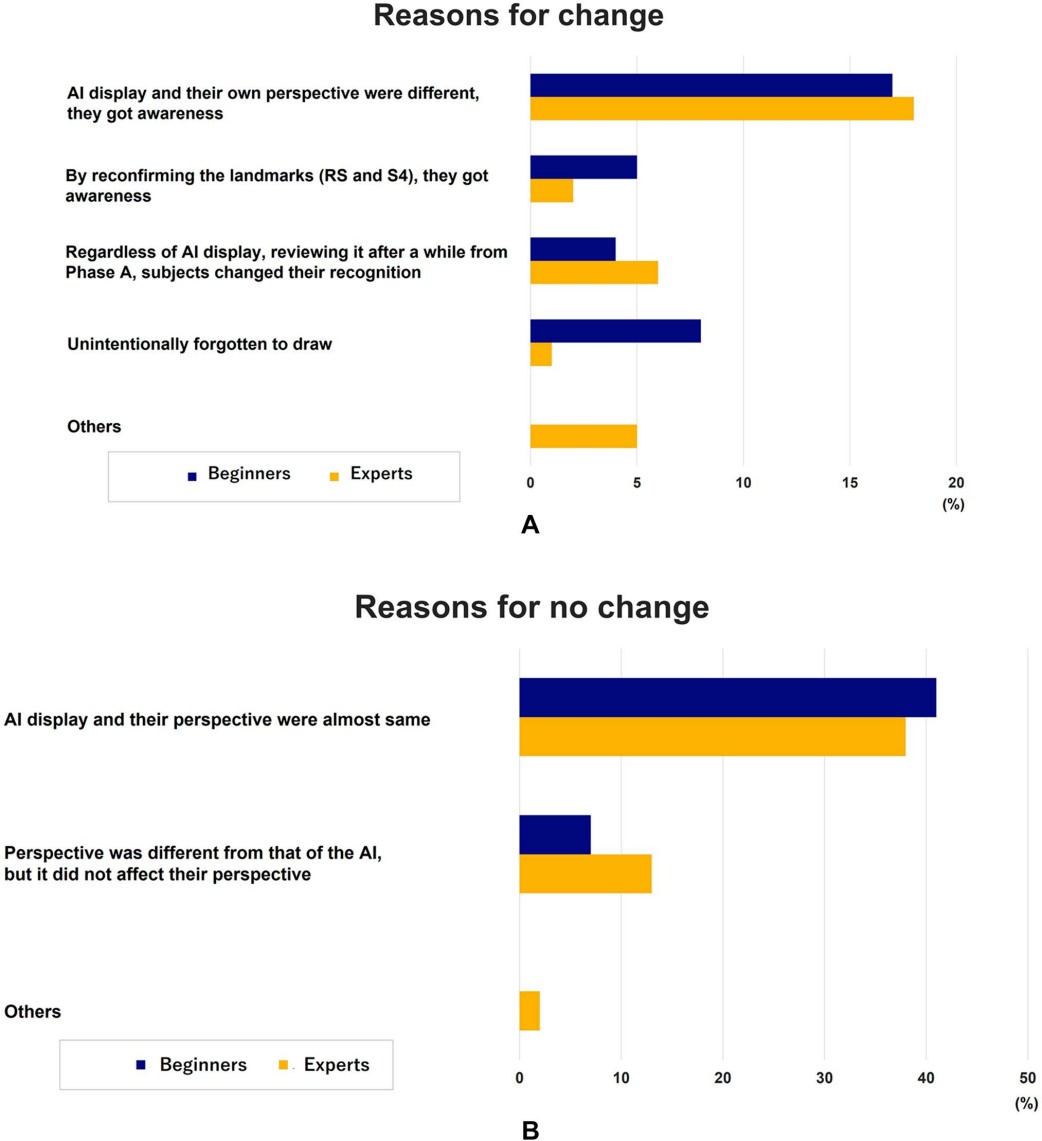
**A** The reasons for change after viewing a 20-s short movie. **B** The reasons for no change after viewing a 20-s short movie

**Fig. 4:**
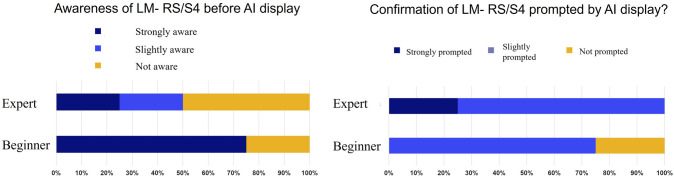
Change of awareness and confirmation rate of LM-RS and LM-S4 after viewing AI display

After viewing a 20-s video, 26.9% of images (43/160) changed their annotations (Fig. 2), and the reasons are shown in Fig. 3A, B. AI display and the subjects’ perspectives were different; however, an awareness of the landmarks resulted in a change in their annotations. This was the main reason for the change in their annotation. On the other hand, 63% of subjects did not change their perspectives after viewing the AI display because each perspective was almost equivalent. Many subjects were aware of the two landmarks (LM-RS and LM-S4) before viewing the AI display, which was further confirmed by the AI display (Phase B, Fig. 4).

**Fig. 5:**
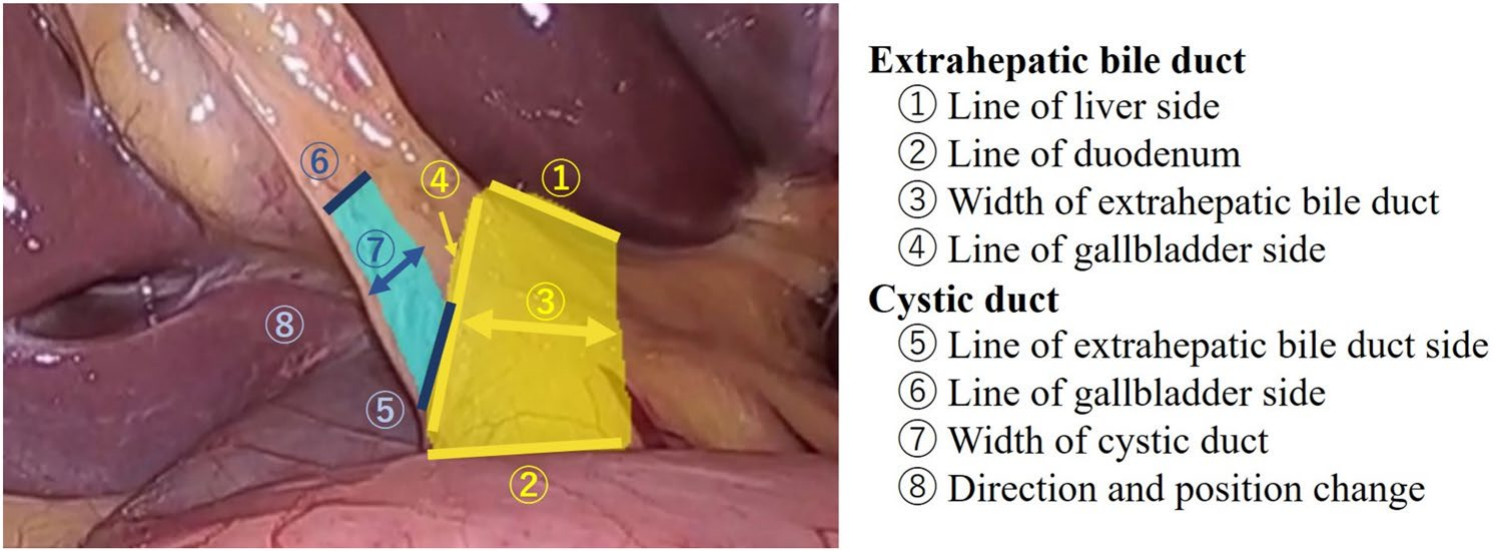
Classification of EHBD and CD

**Fig. 6:**
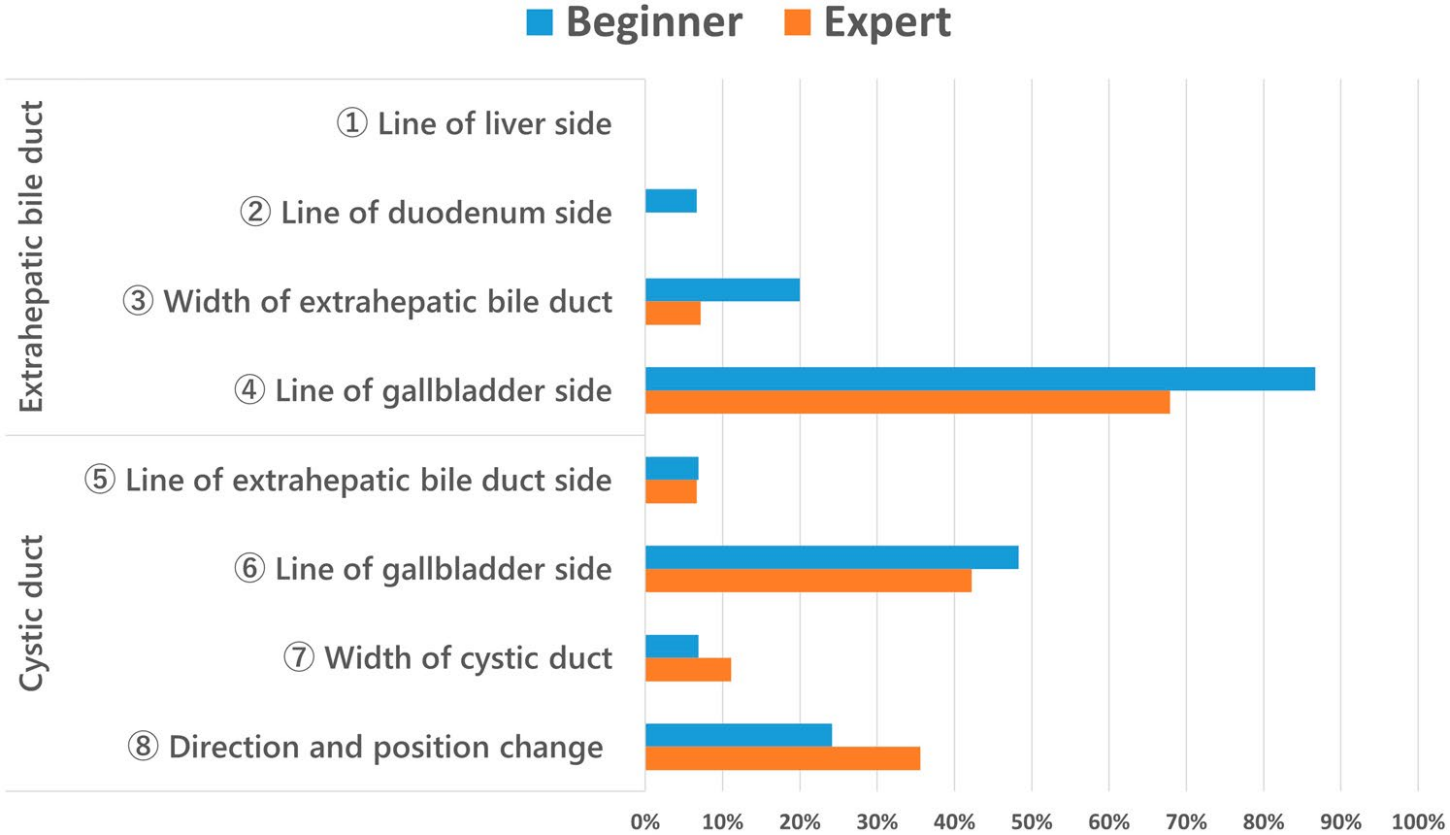
Points to affect BDI prevention

**Fig. 7:**
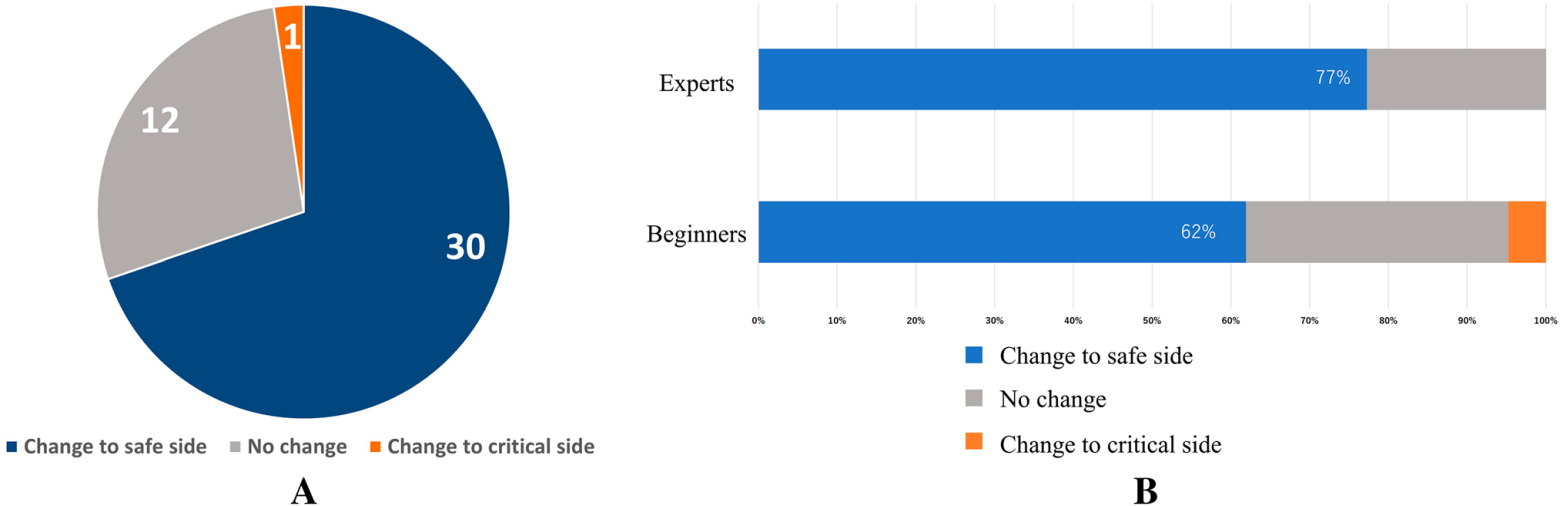
**A** Change of contents. **B** Contents of change between beginners and experts

Four EEC members investigate and contemplated the clinical importance of 43 cases where the subject's perspective changed to a safer or more critical side after viewing the AI display. LM-EHBD and LM-CD were classified into eight parts (Fig. 5), and those most affected by AI were analyzed. According to these results, the shift to the gallbladder side was most frequent in LM- EHBD and LM- CD (Fig. 6). Thirty of the 43 cases (69.8%) were contemplated safer changes, and these changes revealed no differences between starters and experts (62% vs. 77%, respectively; Fig. 7A, B). Of the 27% of annotation changes, 30% were not contemplated to affect the first serosal incision. Only one case (2.3%) revealed that the gallbladder line of EHBD slightly moved to the critical side and contemplated a critical change.

## Discussion

In the present study, the AI display affected subjects’ perspectives by 27%. AI display prompted the recognition of the LM-RS and LM-S4 by both beginners and experts and brought a change of perspective in 70%, contemplating safer changes. This is the first research that clarifies the effect of AI and has illustrated its effectiveness.

Laparoscopic cholecystectomy came into use around 1990. Compared to the past exposed cholecystectomy, LC was much more challenging for the operator to secure visual fields and handle forceps. With advances in medical equipment, LC quickly became the gold standard operation for patients with symptomatic gallstones, simultaneously developing endoscopic equipment [1]. However, BDI, the most severe complication resulting in numerous re-interventions and hospitalizations, has not decreased since 2013 [3].

In the USA, administrative database studies found significant incidences of BDI of 0.15%–0.3% [12–15]. Galli’s database in Sweden contains information on more than 90% of all cholecystectomies performed in that country. The rate of major BDI that required reconstruction was 0.36%, and biliary complications were noted in 1.5% of patients [16]. Information from the National Clinical Database (NCD) system in Japan also reported that the incidence of BDI gradually decreased, but the occurrence of cases has not changed in the last 10 years [3].

Recognizing the CD and cystic artery (CA) was strongly recommended, especially when treating CD. Strasberg et al. suggested the Critical View of Safety in 1995, and the methodology for identifying the CD and CA has since spread throughout the world and is still used today [17]. To avoid BDI in severe cholecystitis, subtotal cholecystectomy, a new surgical strategy, is now widely recognized and used worldwide [18–21]. However, preventing the misidentification of anatomical landmarks due to human error is still difficult, and more action is necessary to solve this problem.

Recently, AI-assisted surgery has been developed for LC. Madani et al. [22] and Laplante et al. [23] created a system that senses safety and danger zones during LC. Mascagni et al. [24] developed a technology to detect the Critical View of Safety (CVS) in surgical images and automatically calculate the CVS score. Golany et al. [25] developed an AI system that identifies surgical phases of simple and complex procedures during LC. However, the evaluation method for assessing the correlation between AI accuracy and clinical needs is unclear. The Dice coefficient is a standard similarity measure between sets commonly used in machine learning [26], but the importance of the Dice coefficient applied to dynamic surgery is unknown. In this study, it was difficult to investigate the Dice coefficient, which is used as an evaluation scale for machine learning. The protocol implemented in this study confirmed the effect of promoting awareness of the S4 and RS when identifying the EHBD and CD landmarks. The beginners understand the significance of being aware of the EHBD and CD more deeply than experts. It was shown that they wanted information to recognize the EHBD and CD that could not be seen directly.

ICG fluorescence methods in LC were used to enhance the localization of CD and EHBD, which may be useful in avoiding BDI [27]. However, there are some disadvantages, such as the rare presence of patients with allergic reactions to ICG administration, the wider than actual emission range of ICG, the limited emission time, and the inability to show the RS and S4 and other important landmarks during LC. On the other hand, the AI system has no impact on the patient and no time limitation, so the complementary action of ICG fluorescence and AI could potentially support the recognition of more accurate anatomical information.

The aim of using AI to teach landmarks is to give awareness of the operator's consciousness toward the landmarks and to provide auxiliary information for accurate landmark recognition. This AI system presents teaching information superimposed on the intraoperative video. AI can analyze the image features of continuous still images input at 30 FPS and draw teaching information in real-time. We can recognize AI's teaching information because the brain's cognitive ability is excellent, but AI finely updates the teaching data even for image features that our naked eyes cannot determine. The Dice coefficient is commonly used as an evaluation scale for machine learning, but it is not suitable for evaluating the performance of AI for superimposed shows on video and the impact of teaching data.

In this study, the activities of AI were intraoperative instruction of landmarks, and the expected effect was to prompt identification of LM-S4 and LM-RS that were necessary to recognize the LM-EHBD and LM-CD, leading to safer first serosal incision before Calot’s triangle dissection. AI increased awareness in 27% of cases; of these, 70% contemplated safe changes. However, of the 27% of annotation changes, 30% were not contemplated to affect the first serosal incision. These changes were primarily reported in EHBD width or duodenal extension and were not contemplated to affect the first serosal incision. Contemplating that the beginning point of the serosal incision is determined dependent on the association between EHBD, S4, and RS, the changes in CD did not have important meaning. Therefore, it was thought that the effect of identifying the CD clearly would avoid damage to the CD itself and would be significant for safe surgery. Only one beginner (2.3%) revealed that the gallbladder line of EHBD slightly moved to the opposite side of the gallbladder and was considered a critical change. The reason for this change was unclear, but it was thought necessary to increase the number of subjects and re-investigate.

This research had many limitations. First, small sample size and investigators. Second, an investigation method for BDI prevention by AI has not been founded. The original use of this AI system was to support interactive recognition of landmarks, but as Pharmaceutical Affairs has not yet approved this system, it is impossible to demonstrate the effects of such usage. Therefore, to evaluate how AI teaching information affects landmarks identified, we could not exceed this stage as a methodology. In this study, however, we implemented the protocol and affirmed the effect of increasing awareness of the LM-S4 and LM-RS when identifying the LM-EHBD and LM-CD by viewing the AI.

In this study, AI provided important awareness for both beginners and experts, prompting them to recognize anatomical landmarks related to reducing BDI. AI was effective in determining a safe serosal incision in this research, and this system may be effective in avoiding BDI. Further research is necessary to clarify that AI systems could avoid BDI.

## Supplementary Information

Below is the link to the electronic supplementary material.

Supplementary file1 (MP4 39394 KB)
